# Impact of previous sepsis on the accuracy of procalcitonin for the early diagnosis of blood stream infection in critically ill patients

**DOI:** 10.1186/1471-2334-8-163

**Published:** 2008-12-02

**Authors:** Pierre Emmanuel Charles, Sylvain Ladoire, Aurélie Snauwaert, Sébastien Prin, Serge Aho, André Pechinot, Niels-Olivier Olsson, Bernard Blettery, Jean-Marc Doise, Jean-Pierre Quenot

**Affiliations:** 1 Service de Réanimation Médicale, Hôpital Le Bocage, C.H.U. de Dijon, France; 2 Service d'Epidémiologie et d'Hygiène Hospitalière, Hôpital Le Bocage, C.H.U. de Dijon, France; 3 Laboratoire d'Immunologie, Hôpital Le Bocage, C.H.U. de Dijon, France; 4 Laboratoire de Bactériologie, Hôpital Le Bocage, C.H.U. de Dijon, France

## Abstract

**Background:**

Blood stream infections (BSI) are life-threatening infections in intensive care units (ICU), and prognosis is highly dependent on early detection. Procalcitonin levels have been shown to accurately and quickly distinguish between BSI and noninfectious inflammatory states in critically ill patients. It is, however, unknown to what extent a recent history of sepsis (namely, secondary sepsis) can affect diagnosis of BSI using PCT.

**Methods:**

review of the medical records of every patient with BSI in whom PCT dosage at the onset of sepsis was available between 1^st ^September, 2006 and 31^st ^July, 2007.

**Results:**

179 episodes of either primary (*n *= 117) or secondary (*n *= 62) sepsis were included. Procalcitonin levels were found to be markedly lower in patients with secondary sepsis than in those without (6.4 [9.5] vs. 55.6 [99.0] ng/mL, respectively; *p *< 0.001), whereas the SOFA score was similar in the two groups. Although patients in the former group were more likely to have received steroids and effective antibiotic therapy prior to the BSI episode, and despite a higher proportion of candidemia in this group, a low PCT value was found to be independently associated with secondary sepsis (Odd Ratio = 0.33, 95% Confidence Interval: 0.16–0.70; *p *= 0.004). Additional patients with suspected but unconfirmed sepsis were used as controls (*n *= 23). Thus, diagnostic accuracy of PCT as assessed by the area under the receiver-operating characteristic curves (AUROCC) measurement was decreased in the patients with secondary sepsis compared to those without (AUROCC = 0.805, 95% CI: 0.699–0.879, vs. 0.934, 95% CI: 0.881–0.970, respectively; *p *< 0.050).

**Conclusion:**

In a critically ill patient with BSI, PCT elevation and diagnosis accuracy could be lower if sepsis is secondary than in those with a first episode of infection.

## Background

When the different causes of intensive care units (ICU)-acquired infections are considered, blood stream infection (BSI) is of particular interest given the reported high rates of associated mortality [1]. Early recognition of such episodes is therefore critical in order to improve outcome in critically ill patients with positive blood cultures [2]. Besides clinical signs and symptoms, other markers that warn the physician about the risk of current systemic infection are required. Among the available biomarkers, serum procalcitonin (PCT) has been described as one of the most promising predictors of bacterial sepsis, especially in the setting of bacteremia [3-6]. Moreover, it has been shown that the degree of PCT elevation was closely related to the degree of organ dysfunction and the outcome in such patients [7-9]. However, it is worth noting that the usefulness of PCT for the diagnosis of sepsis is still a controversial issue as illustrated by a recently published meta-analysis [10]. Actually, the diagnosis accuracy of PCT could vary according to the source of infection and the patient type (i.e., medical vs. surgical) [11,12]. However, it is not clear whether the time between admission to the ICU and the onset of sepsis influences PCT behavior. Obviously, the studies which provide the best predictive values have included almost exclusively patients with sepsis on admittance [7,13]. Such findings are in contrast with those obtained from studies including a larger number of patients with ICU-acquired infections [8,14]. Such patients might be different from the former since they have been exposed to numerous factors likely to influence PCT release, including a previous history of sepsis.

Accordingly, it has been demonstrated in critically ill patients that sepsis was followed by deep alterations in the systemic immune response [15-17]. The peripheral blood monocytes of such patients have been shown to release smaller amounts of inflammatory mediators, especially Tumor Necrosis Factor-α (TNF-α), if exposed to lipopolysaccharide (LPS) in *in vitro *stimulation [18]. This so-called "immune paralysis", could account for an increased risk of secondary infection. Since TNF-α is one of the main determinants of PCT production in response to microbial challenge, we hypothesized that the pattern of PCT elevation was different in critically ill patients who experienced secondary sepsis compared to those in whom it was the first episode of infection.

Every BSI episode and its main characteristics are prospectively recorded in our ICU. Since PCT dosage is also usually performed in every patient with suspected sepsis, this issue was addressed through a retrospective cohort study.

## Methods

### Review of medical charts

Each medical chart was reviewed by a member of the medical staff (AS), unaware of the purpose of the study, following a normalized recording sheet.

### Definitions

One episode of BSI was defined as the recovery of any bacterial or fungal species, in one or more blood cultures. Patients with *Staphylococcus *non-*aureus *bacteremia were not eligible except if at least 2 consecutive samples grew for the same species harboring the same antibiotic resistance pattern.

The onset of sepsis was defined as the time when the first positive blood culture was drawn. Blood samples were obtained by blood punctures before being processed using the BACTEC system based both on standard aerobic and anaerobic media coupled with the 9240 automate (Beckton Dickinson Diagnostic Instrument System, Paramus, NJ, USA). Bacteria and fungi identification was based on standard methods. The onset of BSI was defined as the day when the first positive blood culture was obtained. Two distinct episodes of BSI were considered in one patient if at least 6 days had elapsed between the 2 sets of positive blood cultures, provided appropriate therapy was implemented and significant clinical improvement was obtained between the two episodes. This time-interval was chosen since previously published data indicate that blood culture negativation is obtained in a median time of around 2 days in patients with BSI receiving appropriate antimicrobial treatment.

Sepsis was considered secondary if a proven infection has been documented within a 30-day period prior to the current episode, regardless of its severity, provided the first episode had resolved (i.e., resolution of the clinical symptoms and evidence of clearance of the causative pathogen). Otherwise, sepsis was considered as primary.

### Study population

At least one PCT dosage is routinely performed in our ICU in every patient with systemic inflammatory response syndrome (SIRS), as defined by the American College of Chest Physicians/Society of Critical Care Medicine Consensus Conference, before antimicrobial treatment is started.

Patients admitted to our 15-bed medical ICU between 1^st ^September, 2004 and 31^st ^July, 2007 were eligible if at least one episode of BSI had occurred. The main patient data including the type of admission (admission was considered surgical in patients who had undergone surgery within the 30 days preceding the onset of BSI, and medical otherwise), clinical status at the onset of BSI (i.e., sepsis, severe sepsis or septic shock), the biological findings (including PCT, C-Reactive Protein [CRP] and White Blood Cell [WBC] count), organ dysfunction expressed by the Sepsis-related Organ Failure Assessment (SOFA) score, antibiotics received within the 48 hours preceding the BSI episode, the pathogen(s) involved as well as the infection source if known, were prospectively collected. Treatment with steroids within the 7 days preceding the onset of the sepsis was also taken into account. This was considered significant if the patient had received at least 2 days of treatment with 1 mg/kg or more during the 7 days.

Only patients with at least one PCT dosage obtained within a 12-hour window surrounding the time when the first positive blood culture was drawn were included in the study population. The BSI episodes were then divided into 2 groups according to the kind of sepsis (i.e., secondary or primary).

Another population was analyzed over the last 10 months of the study period (from 1^st ^September, 2006 to 31^st ^July, 2007). Thus, patients undergoing mechanical ventilation for more than 2 days with clinical suspicion of ventilator-associated pneumonia (VAP) as defined by the presence of at least 2 of the following criteria: fever > 38.2°C or hypothermia < 36.5°C, new purulent tracheal aspirates, new lung infiltrate on the chest X-ray, unexplained deterioration in gas exchange, were prospectively enrolled in an observational study that aimed to evaluate the effects of implementing local guidelines for the diagnosis and the management of VAP. This study was approved by the Local Ethic Committee. Notably, PCT dosage was performed in all of these patients at the onset of VAP symptoms as part of the study protocol. Only patients in whom the diagnosis of VAP was considered unlikely (i.e., those with a clinical pulmonary infection score less than 5, regardless of the PCT level), in the absence of another source of infection, were kept for the present study since they constituted a control group of patients with unconfirmed sepsis.

### Measurement of PCT level

The Kryptor^® ^immunoassay was used according to the manufacturer's instructions (Brahms, Hennigsdorf, Germany). The functional sensitivity of the assay is 0.06 ng/mL. Patients in whom PCT was not measured within the 12 hours following the blood sampling were excluded from further analysis because of the risk of false-negative result.

### Statistical analysis

Values are expressed as mean ± SD unless otherwise stated. Continuous variables were compared with the Mann Whitney U test. Categorical variables were compared using the Chi2-test. We then examined the independent contribution of factors that were associated with the "secondary sepsis" criteria in univariate analysis. Because of the non-parametric distribution of the variables of interest, a robust variance estimation was performed prior to logistical regression [19]. In addition, conformity with the linear gradient of each continuous variable was checked. If the linear model did not correspond to the variations, the variable was transformed according to the parsimonious rule. As a result, the log_10_PCT was considered instead of the PCT. The candidate variables were then manually entered into a logistical regression model if the associated regression coefficient had a *p *value less than 0.20 in univariate analysis, and then removed if a *p *value less than 0.05 was obtained by multivariate analysis. The appropriateness of the model was eventually tested.

The diagnosis accuracy of serum PCT levels for the early diagnosis of BSI was then expressed as the area under the corresponding receiver operating characteristic curve (AUROCC) and compared for the two groups (i.e., primary and secondary sepsis) using the values obtained in the control group.

A *p *value < 0.05 was considered as statistically significant for all analyses. The STATA software was used for all analyses (STATA Statistical Package, College Station, Tex., USA).

## Results

### Study population characteristics

Over the 35-month study period, 199 BSI episodes in 194 patients admitted to our ICU were recorded. Among these episodes, 20 were excluded because of the lack of available PCT measurement at the onset of BSI (not done in time, *n *= 19; done in time but delayed analysis of the sample, *n *= 1). As a result, 179 BSIs episodes in 161 patients were retained for final analysis. Main baseline characteristics of the patients with BSI are presented in Table 1. It is worth noting that no difference was found regarding age, gender, type of admission (i.e., medical or surgical) and SAPS II. Among these episodes, 117 were considered primary sepsis as defined previously, whereas the 62 remaining cases were secondary sepsis. The baseline characteristics of all these patients were found to be comparable (Table 1).

**Table 1:** Baseline characteristics and outcome of the patients with blood stream infection according to the sepsis type (i.e. primary or secondary sepsis).

Mean (SD) or number (%)	Primary sepsis *n *= 117	Secondary sepsis *n *= 62	*p*
Age (year-old)	64.9 (14.9)	65.8 (14.0)	0.690

Female/Male	49 (41.9)/68 (58.1)	25 (40.3)/37 (59.7)	0.966

Medical/surgical admission	104 (88.9)/13 (11.1)	49 (79.0)/13 (21.0)	0.119

SAPS II	47.9 (19.3)	49.1 (18.6)	0.673

ICU mortality	34.2%	46.8%	0.137

SAPS: Simplified Acute Physiologic Score; ICU: Intensive Care Unit.

In the patients with secondary sepsis, the diagnosis of the first infection episode was made during a previous hospitalization or within the same ICU stay. The median time elapsed between this first episode and the secondary sepsis was 12.5 day (range: 3–30 day). Septic shock was present in 58.1% of the cases. The main identified bacterial agents were *E. coli *(17.7%), *S. aureus *(16.1%), and *P. aeruginosa *(12.9%). Bacteremia was present in 45.2% of the cases.

Finally, mortality in the ICU was higher in patients with secondary sepsis than in those without, but the difference did not reach statistical significance.

As detailed previously, 23 patients in whom the clinical suspicion of VAP remained unconfirmed, with no evidence of another source of infection, were chosen as negative controls (i.e., patients with infection signs but without sepsis). These patients were not found to be different from those with BSI regarding age, gender, SAPS II and time elapsed from ICU admission to sepsis suspicion (data not shown). However, the SOFA score was more elevated in the patients with BSI than in those without proven sepsis (7.0 [4.0] vs. 5.3 [3.1], respectively; p < 0.05).

### Bloodstream infections

The main characteristics of the included BSI episodes are detailed in Table 2. As expected, secondary sepsis occurred later during the ICU stay than did primary sepsis, and it was more likely to be nosocomial and acquired in the ICU. Exposure to antibiotic therapy that had proved effective *in vitro *against the causative pathogen and to steroids was also more frequent in patients with secondary sepsis. Other differences were noted regarding the causative pathogen and the infection source. BSI occurring as secondary sepsis were more likely to be caused by gram positive bacteria and *Candida *spp than were primary sepsis BSI. In addition, while pneumonia was the most common infection source in both groups, urinary tract infections were significantly less frequent in patients with secondary sepsis than in those without. With regard to the severity of the sepsis, it is worth noting that no difference was found between the two groups in terms of SOFA scores or in the proportion of septic shock. However, although the difference does not reach statistical significance, creatininemia was considerably higher and the platelet count lower in patients with primary sepsis than in those with secondary sepsis BSI.

**Table 2:** Description of the blood stream infection episodes at the time of the first positive blood culture drawing according to the sepsis type (i.e. primary or secondary sepsis).

Mean (SD) or number (%)	Primary sepsis *n *= 117	Secondary sepsis *n *= 62	*p*
Time elapsed from ICU admission (N. of days)	2.0 (2.4)	15.6 (16.9)	< 0.0001

SOFA score	6.9 (4.1)	7.2 (3.9)	0.706

Platelet count (cell/mm3)	185,459 (118,375)	235,935 (183,485)	0.028

Creatininemia (μmol/L)	198.7 (168.5)	163.2 (129.2)	0.149

Prothrombin time (%)	58.8 (21.9)	62.8 (19.9)	0.248

Septic shock	60 (51.7)	31 (50.0)	0.302

Nosocomial sepsis ICU acquired sepsis	34 (35.8) 31 (26.5)	62 (100) 52 (83.9)	< 0.0001 < 0.0001

Infection source			0.002
Lung	30 (25.6)	12 (19.3)	0.447
Abdominal	23 (19.7)	18 (29.0)	0.217
Soft tissues	13 (11.1)	4 (6.4)	0.456
Urinary tract	24 (20.5)	2 (3.2)	0.004
Miscellaneous	9 (7.7)	5 (8.1)	0.480
Unknown	18 (15.4)	21 (33.9)	0.008

Involved pathogen bacteria	113 (95.6)	41 (64.4)	< 0.0001
Gram positive	51 (43.6)	20 (32.2)	
Gram negative	62 (53.0)	21 (33.9)	
fungi			
*Candida *spp	4 (3.4)	21 (33.9)	

Previous exposure to antibiotic therapy with *in vitro *activity against the involved pathogen	16 (13.7)	21 (33.9)	0.003

Previous exposure to steroids	9 (7.7)	23 (37.1)	< 0.0001

Time elapsed between bacteremia onset and PCT sampling (N. of minutes)			
Mean (SD)	-169.7 (382.6)	6.3 (455.4)	0.011
Median [range]	-13.0 [-1072–720]	0.0 [-720–720]	

ICU: Intensive Care Unit; SOFA: Sepsis-related Organ Failure Assessment.

### Serum PCT, CRP and WBC measurements

**Figure 1:**
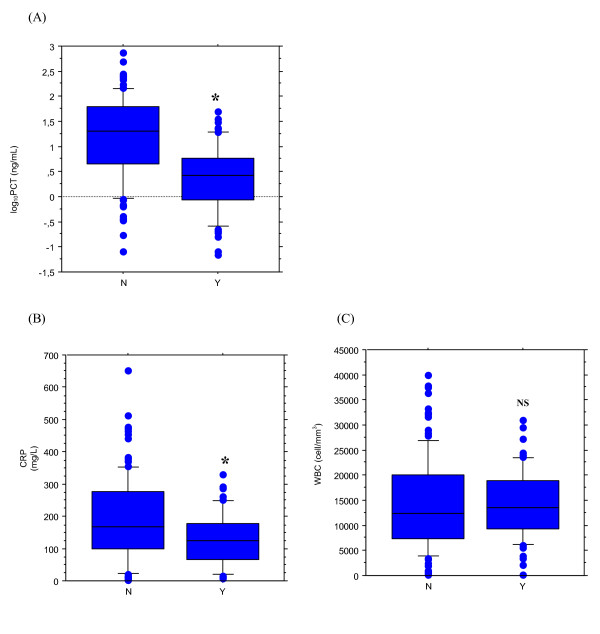
**Serum procalcitonin (PCT) level (*Fig*. A), white blood cells count (WBC) (*Fig*. B) and C-reactive protein (CRP) level (*Fig*. C), at the onset of blood stream infection according to its primary (*left boxes*, *n *= 127) or secondary status (*right boxes*, *n *= 62) in critically ill patients with clinical sepsis.** Data are presented as box plots with median lines, 25- and 75-percentile boxes, and 10- and 90-percentile error bars. The circles represent the outliers. A log scale is used for the Y-axis in Fig. A. * indicate *p *< 0.05 between primary and secondary sepsis.

Serum procalcitonin was significantly lower in patients with secondary sepsis than in those without (6.4 [9.5] vs. 55.6 [99.0] ng/mL, respectively; *p *< 0.001) (Figure 1). The difference was markedly less pronounced for CRP (128.2 [83.1] vs. 188.3 [129.6] mg/L; *p *= 0.002), while white blood cell count was comparable (14305 [6885] vs. 14376 [9209] cell/mm^3^; *p *= 0.958).

Since secondary infections were more likely to be ICU-acquired, we hypothesized that blood samples used to determine PCT levels were obtained earlier during the course of sepsis than were those in the case of primary sepsis. The time between the onset of sepsis and the sampling for PCT dosage was therefore measured. Although the median time in the two groups was comparable, PCT was obtained significantly earlier in the patients with primary sepsis than in those with secondary sepsis (Table 2).

The variables found to be associated with secondary sepsis in univariate analysis were entered into a multivariate analysis model (Table 3, top). Thus, a lower level of PCT was found to be independently associated with secondary sepsis (Odd Ratio = 0.33, 95% CI: 0.16–0.70; *p *= 0.004), as was the time from ICU admission, candidemia and the exposure to effective antibiotic therapy prior to BSI. Given the fact that PCT elevation is thought to be lower in the patients with candidemia than in those with bacteremia, a second multivariate analysis model was built excluding the former episodes of BSI. The same independent predictors of secondary sepsis were identified as in the previous model (Table 3, bottom).

**Table 3:** Multivariate analysis of the factors associated with secondary sepsis in critically ill patients with blood stream infection.

	Odd ratio	Variable type	95% CI		*p*
Time elapsed from ICU admission	1.38	continuous	1.23	1.54	< 0.001

Log_10_PCT	0.33	continuous	0.16	0.70	0.004

Candidemia (Yes)	7.02	dichotomous	1.48	33.2	0.014

Previous exposure to antibiotic therapy with *in vitro *activity against the involved pathogen (Yes)	4.28	dichotomous	1.31	13.95	0.016

ICU: Intensive Care Unit; PCT: Procalcitonin; CI: Confidence Interval.

### Accuracy of PCT as a diagnostic tool

Given the markedly lower magnitude of PCT elevation in the context of secondary sepsis than in patients with primary sepsis, we sought to compare its value as a diagnostic tool in the two situations. The abovementioned population of patients with SIRS and unconfirmed suspicion of VAP was used as control and the corresponding ROC curves were constructed.

**Figure 2:**
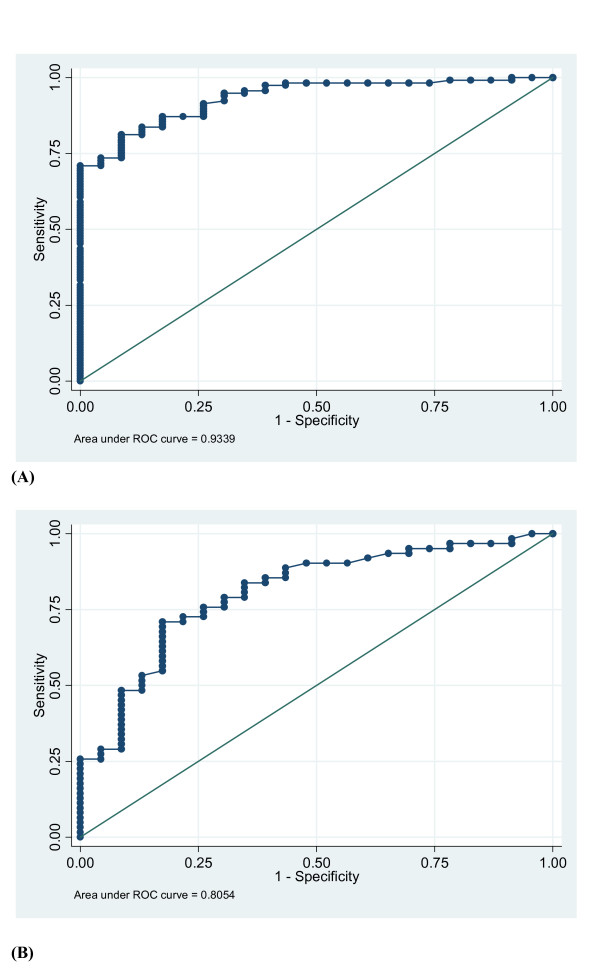
**Receiver operating characteristic (ROC) curve of serum procalcitonin (PCT) for the diagnosis of blood stream infection in critically ill patients with either primary (*Fig*. A), or secondary (*Fig*. B) sepsis.** Plain circles indicate PCT values. Area under the ROC curve = 0.934, 95% CI: 0.881–0.970; vs. 0.805, 95% CI: 0.699–0.879, respectively; *p *< 0.050.

Hence, we found that the accuracy of PCT in the diagnosis of BSI in patients with secondary sepsis, as assessed through the area under the ROC curve (AUROCC) calculation, was not as good as in those with primary sepsis (0.934, 95% CI: 0.881–0.970; vs. 0.805, 95% CI: 0.699–0.879, respectively; *p *< 0.050) (Figure 2).

Finally, we sought to determine the best PCT cut-off values to differentiate between SIRS and sepsis in our two groups of patients. Obviously, reliable identification of patients with secondary sepsis required lower values than in those with primary sepsis (Table 4). Thus, the sensitivity of PCT reached 83.8% in the patients with primary sepsis if the 2.0 ng/mL threshold was applied, whereas it did not exceed 58% in those with secondary sepsis. As a result, the negative predictive value was found to be markedly lower in these latter patients, even if the 0.25 ng/mL threshold value was used.

**Table 4:** Diagnostic accuracy of serum PCT for the diagnosis of sepsis in critically ill patients with either primary or secondary BSI-related sepsis.

PCT cutoff value (ng/mL)	Sepsis type	Sensitivity (%)	Specificity (%)	Positive predictive value (%)	Negative predictive value (%)
PCT ≥ 0.25	Primary	98.3	47.8	90.5	84.6
	Secondary	90.3	47.8	82.3	64.7

PCT ≥ 0.50	Primary	94.9	65.2	93.3	71.4
	Secondary	83.9	65.2	86.7	60.0

PCT ≥ 1.00	Primary	89.7	73.9	94.6	58.6
	Secondary	72.6	73.9	88.2	50.0

PCT ≥ 2.00	Primary	83.8	82.6	96.1	50.0
	Secondary	58.1	82.6	90.0	42.2

PCT: procalcitonin; BSI: blood stream infection.

## Discussion

We show here that PCT elevation at the onset of sepsis is lower in patients with secondary sepsis-related BSI than in those experiencing their first episode of systemic infection, regardless of the severity of the disease. In addition, the accuracy of PCT in the diagnosis of BSI could be decreased in this subset of critically ill patients, especially if the negative predictive value of the test was considered. Such findings could account for the apparent lack of reliability of PCT for the diagnosis of ICU-acquired sepsis [20,21].

Since PCT elevation reflects the inflammatory cytokine response to one bacterial insult, such a difference could be explained by the so-called immune paralysis paradigm. We cannot, however, draw any definite conclusions regarding this point since cytokine levels were not measured simultaneously with PCT. Further prospective studies are therefore needed to test this hypothesis.

Another explanation is the fact that in our study, secondary sepsis was more likely to be caused by gram positive bacteria and *Candida *species than was primary sepsis. Actually, we have shown previously, like others, that PCT elevation could be lower when these pathogens are isolated from blood cultures as compared to gram negative bacteria [22-24]. In addition, a low PCT elevation was still found as an independent predictor of secondary sepsis after having excluded from the analysis the patients with candidemia.

The medication given to patients prior to sepsis could also account for differences in PCT elevation. Since patients in the secondary sepsis group spent more time in the ICU than did those without, one could expect that they were more likely to have received steroids and antibiotics prior to the septic episode we analyzed. We did in fact find that larger amounts of steroids had been given to them. Similarly, patients with secondary sepsis were more likely to have received at least one antibiotic agent active against the causative bacteria prior to sepsis. Taken together, these data suggest that the inflammatory response as reflected by PCT elevation might have been blunted by steroids and/or appropriate antibiotics. However, although cumulative evidence has emphasized the anti-inflammatory impact of steroids in critically ill patients, there is little clinical evidence of a possible relationship with PCT elevation during sepsis [25-28]. The impact of prior treatment with antibiotics is also debatable. According to *in vitro *observations, bactericidal antibiotic therapy should accentuate the inflammatory response rather than blunt it through the liberation of microbial products that are known to trigger the release of PCT [29]. As a result, our findings provide putative but questionable explanations regarding the differences between secondary and primary sepsis in terms of PCT elevation.

Several limitations of our study must be mentioned. Because of the retrospective design, putative confounding variables cannot be excluded. PCT dosing may not have been performed within the same time frame in both groups. In accordance with this possibility, but surprisingly, the mean time between the onset of sepsis and the taking of blood samples for PCT analysis was longer in patients with secondary sepsis than in those without. We should therefore consider this difference as a conservative bias since higher values should therefore have been expected in the secondary sepsis group. In addition, similar results in terms of PCT elevation were obtained when only patients with ICU-acquired sepsis were considered (data not shown). Finally, one cannot exclude the possibility that despite comparable SAPS II on admission and SOFA scores patients with primary sepsis were sicker than those with secondary sepsis since higher levels of creatininemia and lower platelet counts were reported. This finding as well as renal impairment *per se *could account for greater PCT elevation in these patients [8,30]. In addition, it is known that a longer ICU stay is associated with a worse nutritional status, which could in turn lead to a lower PCT release, although evidence is still lacking [31]. However, the fact that mortality was lower in patients with primary sepsis makes this hypothesis unlikely. The choice of our control group could also raise several concerns. Hence, it is worth noting that the SOFA score was lower in this group than in the patients with proven sepsis (i.e., BSI). This could have led to an overestimation of the diagnosis accuracy of PCT in differentiating between sepsis and non-infectious inflammatory states. This was however not the objective of our study. In addition, one could also argue that patients from the control group might have met S.I.R.S. criteria several days before VAP suspicion. This could account for an immunodepression state and in turn, for lower PCT values [32]. Any way, such limitations do not influence the comparison between the patients with secondary sepsis to those without.

## Conclusion

Despite the possible shortcomings, we have shown that PCT might be less reliable for the diagnosis of sepsis in critically ill patients with secondary sepsis than in those with a first episode of BSI. Since milder PCT elevation is expected in the former patients, it may be advisable to use lower cut-off values in the setting of secondary sepsis. In addition, our findings emphasize the impact of factors such as time from ICU admission or previous exposure to an effective antibiotic therapy on PCT elevation. These should be therefore taken in account in any study that aims to determine the accuracy of PCT in the diagnosis of BSI in critically ill patients.

## Abbreviations

BSI: blood stream infection; ICU: intensive care unit; PCT: procalcitonin; WBC: white blood cell count; CRP: C-reactive protein; AUROCC: area under the receiver operating characteristic curve; CI: confidence interval; SAPS: simplified acute physiologic score; SOFA: sepsis-related organ failure assessment; VAP: ventilator associated pneumonia.

## Competing interests

PEC has received payments from Brahms (Hennigsdorf, Germany) to attend one meeting about sepsis management. The other authors have not received any payment.

## Authors' contributions

PEC designed the study, analyzed the data and drafted the manuscript. SL and AS collected the data and participated to their interpretation. SA performed the statistical analysis. SL, JPQ, JMD, SP and BB participated to the redaction of the manuscript. NOO managed the activity of the Immunology Laboratory. AP managed the activity of the Bacteriology Laboratory.

## Pre-publication history

The pre-publication history for this paper can be accessed here:


